# In Vitro and In Silico Analysis of Antioxidant and Antifungal Activity of Essential Oil From *Achillea biebersteinii*

**DOI:** 10.1002/cplu.70246

**Published:** 2026-09-22

**Authors:** Zehra Torun, Tuba Unver, M. Abdullah Alagoz

**Affiliations:** ^1^ Department of Pharmacognosy Faculty of Pharmacy Inonu University Malatya Turkiye; ^2^ Department of Pharmaceutical Microbiology Faculty of Pharmacy Inonu University Malatya Turkiye; ^3^ Department of Pharmaceutical Chemistry Faculty of Pharmacy Inonu University Malatya Turkiye

**Keywords:** α‐terpineol, *Achillea biebersteinii*, antioxidant, antifungal, molecular modeling, *trans*‐Pinocarveol

## Abstract

This study aims to ascertain the phytochemical composition of the essential oil obtained from *Achillea biebersteinii* Afan. flowers, to validate its antioxidant and antifungal properties, and to identify the active components implicated in the antifungal mechanisms by molecular docking analysis. The 46 different compounds were identified by GC–MS analysis, 14 of which were present at concentrations greater than 1%. The main compounds are eucalyptol (21.84%), *cis*‐Chrysanthenol (17.86%), camphor (15.29%), (−)‐α‐Pinene (6.14%), *trans*‐Chrysanthenyl acetate (5.80%). The DPPH• and ABTS^+^ radical scavenging effects of the essential oil were shown be moderately active, with IC_50_ values of 11.76 and 3.45 mg/mL. As a result of antifungal activity, *A. biebersteinii* flower essential oil demonstrated substantial activity on *Candida* species. The MIC of the essential oil against *C. glabrata* and *C. parapsilosis* was 0.937 µL/mL, while against other *Candida* species it was 3.75 µL/mL. Molecular docking was carried out to determine the effects of compounds in the essential oil of *A. biebersteinii* flowers on antifungal activity. In silico studies showed that α‐Terpineol and *trans*‐Pinocarveol, with docking scores, were two of the components with the highest affinity against both *C. glabrata* and *C. parapsilosis* and were determined to be potential antifungal compounds.

## Graphical abstract

Essential oil of *Achillea biebersteinii* Afan. flowers exhibits moderate antioxidant activity in DPPH• and ABTS^+^ assays, while it demonstrates high anticandidal activity, with MFC (0.937−3.75 μL/mL) values. α‐Terpineol and trans‐Pinocarveol are antifungal compounds responsible for the effect against both *C. glabrata* and *C. parapsilosis* and are shown through molecular docking.

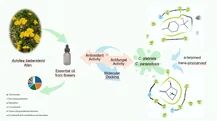

## 1 Introduction

Aromatic and medicinal plants have been utilized as pioneering treatments from ancient times to the present. Aromatherapy treatment is fundamentally based on essential oils, which positively impact human health both spiritually and physically [1, 2]. Numerous plants within the Asteraceae family, commonly referred to as the daisy family, include substantial concentrations of essential oils. The *Achillea* genus, named after Achilles from Greek mythology and also referred to as Yarrow, is a member of the Asteraceae family and comprises 115 species distributed throughout Europe, Asia, North Africa, North America, Australia, and New Zealand [3, 4]. According to the list of Turkish plants, there are 50 different *Achillea* species [5].

*Achillea biebersteinii* Afan. is an Iranian–Turanian element, a perennial, flowering herbaceous or semi‐shrub plant that is prevalent globally, encompassing Central Asia, Afghanistan, Iran, the Middle East, Southern Russia, Eastern Europe, the Caucasus, and Turkiye [6]. The species, which is reported to have 56 taxa throughout Turkiye, 28 of which are endemic, develops as a perennial plant that can grow to a length of approximately 10–100 cm, has a thick or thin woody rhizome, and occasionally creeps. The plant can be collected in diverse settings such as woodland clearings, rocky inclines, desert grasslands, steppes, field margins, and dry meadows throughout April to May at elevations ranging from 350 to 3450  m [6, 7]. Ethnobotanical surveys [8] in various regions of Turkiye indicated that this plant is mainly used for the treatment of stomachache [9, 10, 11, 12, 13, 14], abdominal pain [15, 16, 17, 18], menstrual pain [11, 16, 19, 20], wound healing, and hemostasis [21, 22, 23], rheumatism, sinusitis, and toothache [24, 25]. The most frequently used plant parts are flowers and aerial parts, mostly as infusions or decoctions, but for wound treatment, locally crushed or mashed preparations are applied [10, 21, 23, 26]. It is used outside Turkiye for indigestion, fever, and hypoglycemia in Iran [27, 28, 29, 30]; to combat leishmaniasis, as an insect deterrent, and for dental pain in Saudi Arabia [31, 32]; and for diarrhea and headaches in Uzbekistan [33].

Phytochemical analyzes demonstrated the high occurrence of phenolic acids in *A. biebersteinii* extracts. Chlorogenic acid was the major component of flowers, leaves, and roots, along with caffeic acid, cynarin, and other caffeoylquinic acid derivatives [34, 35, 36, 37]. Flavonoids like apigenin, luteolin, rutin, quercetin, axillarin, and jaceidin [35, 36, 38, 39, 40, 41], along with sesquiterpene lactones such as micranthin and sintenin [42], monoterpenes including ascaridole, the diterpene strictic acid [43], fatty acids, and the phytosterol *β*‐sitosterol [39], have also been documented [8].

The essential oil of *A. biebersteinii* shows clear quantitative and qualitative differences according to the geographic origin, and this chemotypic variation seems to be related to the reported results on biological activities. In Turkish populations, 1,8‐cineole and camphor were primarily identified as the major constituents [44, 45, 46], whereas piperitone‐dominant chemotypes were distributed in various regions such as Ağrı, Erzurum, and Sivas [47, 48]. Conversely, the ascaridole‐ or cis‐ascaridole‐dominated oils were typical of the populations from Egypt, Jordan, and other parts of Iran [49, 50, 51], reflecting significant chemotypic variations across the geographic distribution of the species [8]. The pharmacological efficiency of *A. biebersteinii* essential oil is significantly affected by its geographical and chemotypic variability, and research relating specific chemotypes to bioactivity findings will clarify the compounds responsible for these effects.

In recent years, the incidence of fungal infections has increased, and the development of antifungal resistance among fungal cells has made clinical treatment more challenging. Fungal cells are the most important cause of morbidity and mortality in immunocompromised and hospitalized patients [52, 53]. *Candida* species are the most common fungal pathogens capable of initiating infection, and the incidence of infection is higher in immunocompromised individuals [54]. *Candida* species are generally asymptomatic commensal organisms that colonize mucosal surfaces but can cause serious life‐threatening infections such as candidemia [52, 55, 56]. Although *Candida albicans* is the most common *Candida* infection, infections caused by non‐*albicans Candida* species have serious consequences [55]. It is known that various factors, such as antibiotic use, disease, immunosuppression, premature birth, and burns, invite candidemia. Non‐*albicans Candida* are generally resistant to antifungals due to their active virulence factors [55, 57, 58]. The most important causative agents of candidemia are *C. albicans*, followed by *Candida tropicalis*, *Candida glabrata*, *Candida krusei*, and *Candida parapsilosis* [59]. Infections caused by *Candida* species are challenging to treat and can be prolonged. Therefore, the application of phytotherapy, which has gained popularity in recent years, in the treatment of candidal diseases is promising.

Although studies are available on the antifungal capacities [60, 61] of the *Achillea biebersteinii* essential oils, no conclusive study exists to determine the compounds based on chemotypic differences in the essential oil composition. In this sense, the present study aimed to identify the compound responsible for the antifungal activity evaluation through in silico molecular docking, filling this gap in the scientific field. We selected two fungal targets addressing distinct mechanisms. The first, lanosterol 14α‐demethylase (CYP51), catalyzes a committed step in ergosterol biosynthesis, and its inhibition disrupts membrane integrity (PDB ID: 5JLC) [62, 63]. The second is the secreted aspartic protease Sapp1p of *C. parapsilosis* (PDB ID: 7AGB) [64], a virulence‐directed target: Saps degrade host defence proteins and promote adhesion and biofilm formation, making them among the best‐characterized virulence factors of non‐*albicans* *Candida*. These targets span a biosynthetic and a virulence‐associated mechanism and match the two species most susceptible in our assays. Here, docking served to reveal plausible binding modes and their structural basis rather than predict absolute affinities. This study intends to determine the phytochemical composition of the essential oil extracted from *A. biebersteinii* flowers, to confirm its antioxidant and antifungal capabilities, and to identify the active components involved in the antifungal mechanisms by molecular docking analysis. This approach provides a basis for future study planning with essential oils containing compounds that fight Candida species and for the development of pharmaceutical products.

## 2 Results

### 2.1 Yield and Chemical Composition of Essential Oil

As a result of hydrodistillation, the essential oil is obtained: 0.45 µL. The essential oil yield from *A. biebersteinii* flowers is 0.87 µL/g. 46 components were discovered, comprising 99.57% of the essential oil. Among these, 14 compounds were observed as the most dominant compounds found at more than 1% v/v (Table 1). These are eucalyptol (21.84%), *cis*‐Chrysanthenol (17.86%), camphor (15.29%), (−)‐α‐Pinene (6.14%), *trans*‐Chrysanthenyl acetate (5.80%), Camphene (4.47%), *p*‐Cymene (3.63%), α‐Chloropinacolone (2.78%), borneol (2.41%), *trans‐*Pinocarveol (1.56%), 3‐Ethyltoluene (1.30%), 3‐Thujanone (1.20%), α‐Terpineol (1.19%), α‐Campholenal (1.14%) respectively. These fourteen compounds are believed to significantly influence the properties of the essential oil, whereas the other 37 compounds, present at concentrations below 1%, may exert a minimal synergistic effect on the essential oil's activities [65].

**TABLE 1:** Phytochemical components of essential oil from *A. biebersteinii* flowers.

Peak/compound number	Compound	Retantion time, min	Peak area, %	Classification
1	3‐Methylapopinene	6.810	0.41	Monoterpen
2	(−)‐α‐Pinene	7.079	6.14	Monoterpene
3	Camphene	7.436	4.47	Monoterpene
4	β‐Pinene	8.099	0.45	Monoterpene
5	6,6‐Dimethylhepta‐2,4‐diene	8.410	0.22	Unsaturated hydrocarbon
6	3‐Ethyltoluene	8.473	1.30	Aromatic hydrocarbon
7	*p*‐Cymene	9.236	3.63	Monoterpene
8	Eucalyptol	9.453	21.84	Monoterpene
9	*γ*‐Terpinene	10.079	0.14	Monoterpene
10	*Trans*‐Linalool oxide	10.415	0.33	Monoterpenoid
11	*Cis‐*Linalool oxide	10.807	0.41	Monoterpenoid
12	β*‐*Linalool	11.052	0.89	Monoterpenoid
13	Hotrienol	11.164	0.92	Tertiary alcohol
14	3‐Thujanone	11.268	1.20	Monoterpenoid ketone
15	β‐Thujone	11.535	0.38	Monoterpene ketone
16	*Cis*‐2‐*p*‐Menthen‐1‐ol	11.642	0.29	Secondary alcohol
17	α‐Campholenal	11.759	1.14	Monoterpenoid
18	(+, −)‐1,3,3‐Trimethylcyclohex‐1‐ene‐4‐carboxaldehyde	12.008	0.47	ND
19	*Trans‐*Pinocarveol	12.143	1.56	Monoterpenoid
20	Camphor	12.299	15.29	Monoterpene ketone
21	Sabina ketone	12.552	0.48	Monoterpene ketone
22	*Cis*‐Chrysanthenol	12.702	17.86	Monoterpenoid
23	Borneol	12.807	2.41	Monoterpenoid
24	Terpinen‐4‐ol	13.053	0.45	Monoterpenoid
25	Cumic alcohol	13.189	0.40	Monoterpenoid
26	α‐Terpineol	13.349	1.19	Monoterpenoid
27	Myrtenal	13.514	0.46	Monoterpene
28	Verbenone	13.823	0.39	Terpene
29	*Cis‐*Carveol	14.000	0.57	Monoterpenoid
30	Cumaldehyde	14.526	0.19	Benzaldehyde
31	*Trans*‐Chrysanthenyl acetate	15.010	5.80	Monoterpenoid
32	Bornyl acetate	15.600	0.59	Monoterpenoid
33	*p*‐Cymen‐7‐ol	15.671	0.39	Monoterpenoid
34	Isoascaridole	16.062	0.14	Monoterpenoid
35	*Trans*‐Carvyl propionate	17.129	0.51	ND
36	Hotrienyl acetate	18.077	0.58	ND
37	*α*‐Chloropinacolone	19.945	2.78	Butanone
38	Benzyl isobutyl ketone	20.010	0.27	Benzene
39	1‐Cyclopentene‐1‐methanol	21.909	0.35	ND
40	5‐(Hydroxymethyl)spiro [2.4]heptan‐5‐ol	22.101	0.50	ND
41	β‐Selinenol	23.349	0.16	Sesquiterpene
42	Patchouli alcohol	23.799	0.15	Sesquiterpene
43	Benzoic acid, hexyl ester	26.519	0.43	Benzoic acid derivetives
44	Phytone	26.580	0.21	Terpene hydrocarbon
45	Benzoic acid, heptyl ester	26.878	0.24	Benzoic acid derivetives
46	Isocyclocitral	27.579	0.19	ND
47	(7,7‐Dimethyl‐2‐oxobicyclo [2.2.1]hept‐1‐yl) methanesulfonic acid, methyl ester	28.747	0.39	Monoterpenoid
	Total		99.57%	

Abbreviation: ND, not dedection.

### 2.2 Antioxidant Activity Result

**FIGURE 1:**
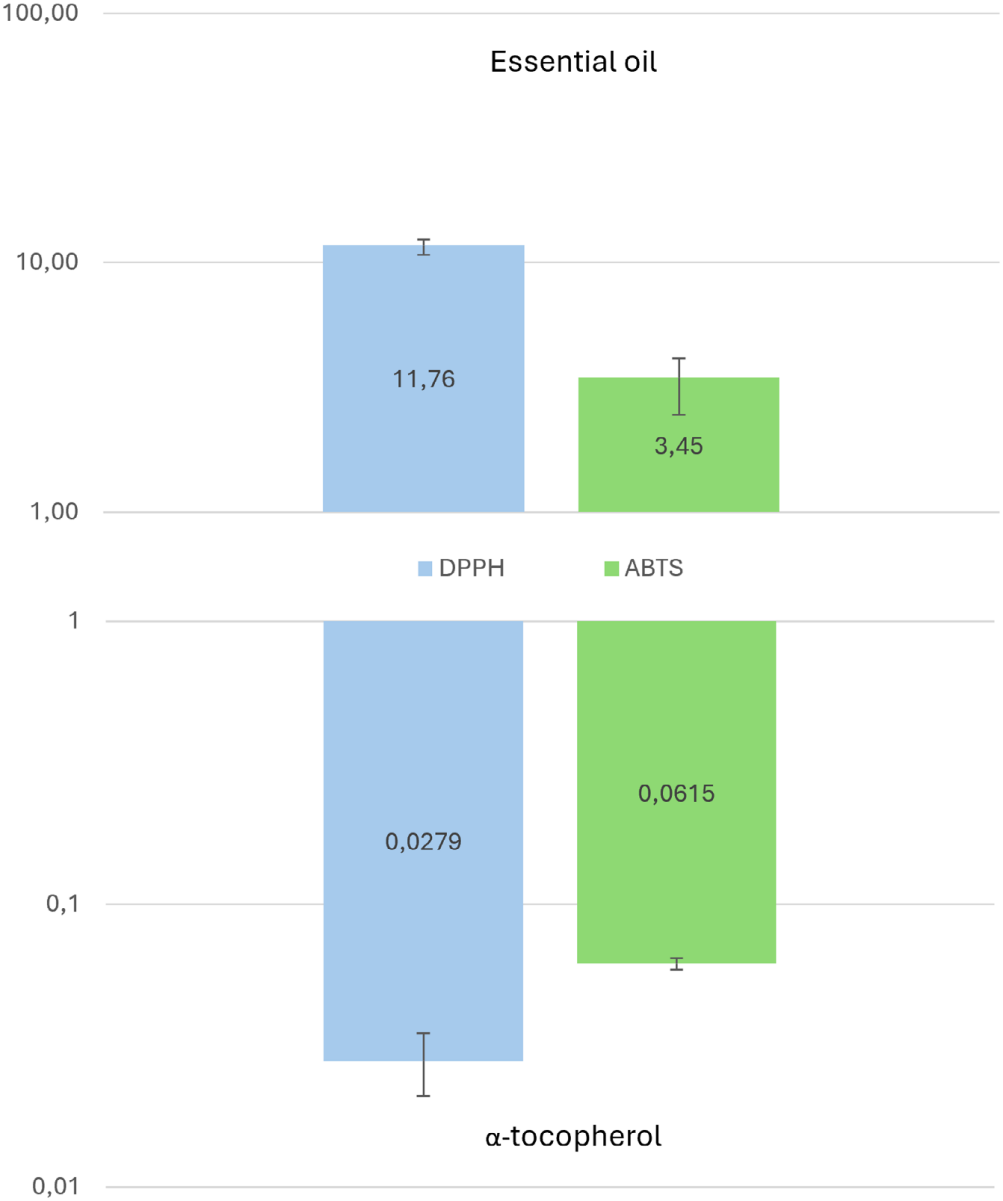
Log‐scale representation of radical scavenging activities of α‐tocopherol and essential oil.

The antioxidant results of the essential oil obtained from *A. biebersteinii* according to the DPPH• and ABTS^+^ methods were evaluated against α‐tocopherol, one of the natural antioxidants, using the independent‐sample *t*‐test. IC_50_ values are expressed for essential oil and α‐tocopherol as µL/mL and mg/mL, respectively. Since the essential oil is liquid, the volume taken was weighed, and the µL/mL conversion was done to obtain mg/mL. The IC_50_ values of essential oil and α‐tocopherol were calculated using calibration equations of *y* = 3.8898*x* + 4.2431 (*R* ^2^ = 0.998) and *y* = 1588.2*x* + 5.6426 (*R* ^2^ = 0.998), respectively, for the DPPH• test and *y* = 3.9505*x* + 5.0235 (*R* ^2^ = 0.9903) and *y* = 863.17*x* − 1.836 (*R* ^2^ = 0.997), respectively, for the ABTS^+^ test. The results are given in Figure 1 and Table 2. The IC_50_ value of the essential oil was compared with α‐tocopherol as the standard using the independent‐sample *t*‐test. Statistically, a significant difference was found with a *p* < 0.05 value.

**TABLE 2:** Antioxidant result of the essential oil of *A. biebersteinii* flowers.

Samples	IC_50_ a			
	DPPH•		ABTS^+^	
Essential oil	38.819 ± 2.00 µL/mL (11.76 ± 0.60 mg/mL)		11.385 ± 2.28 µL/mL (3.45 ± 0.69 mg/mL)	
α‐Tocopherol	0.0279 ± 0.0007 mg/mL		0.0615 ± 0.003 mg/mL	

a Values are presented as mean ± standard deviation (*n* = 3). Results are presented as IC_50_. Statistical analysis was conducted with Student's *t*‐test. Distinct superscript letters within the same column denote significant differences (*p* < 0.05).

### 2.3 Determination of Minimum Inhibitory Concentration

**FIGURE 2:**
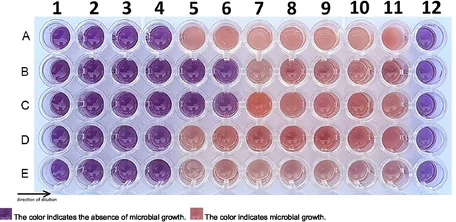
The results of the antifungal activity of essential oil from *A. biebersteinii* flowers using the broth dilution method. (A) *C. krusei*, (B) *C. glabrata*, (C) *C. parapsilosis*, (D) *C. tropicalis,* and (E) *C. albicans*. The essential oil concentrations between the 1st and 10th wells range between 30, 15, 7.5, 3.75, 1.875, 0.938, 0.469, 0.234, 0.117, and 0.058 µL/mL, respectively. Eleventh and twelfth wells are the positive and negative controls, respectively.

According to the broth dilution method results, the color change caused by resazurin, used as an indicator on the microplate, indicates fungal growth in that well [66, 67]. The MIC value is the lowest concentration of an essential oil at which no microbial growth occurs as the concentration decreases. Accordingly, the lowest essential oil concentration at which no color change is observed in the microplate is determined as the MIC. *C. krusei*, *C. tropicalis,* and *C. albicans* inoculated in rows A, D, and E showed growth starting from the fifth well (Figure 2). Therefore, the minimum inhibitory concentration of the essential oil against these microorganisms is 3.75 µL/mL, as determined in the fourth well. *C. glabrata* and *C. parapsilosis*, inoculated into rows B and C on the microplate, started to grow from the seventh well onward. Therefore, the MIC value of the essential oil for these species is 0.937 µL/mL, which is the value in the sixth well.

**FIGURE 3:**
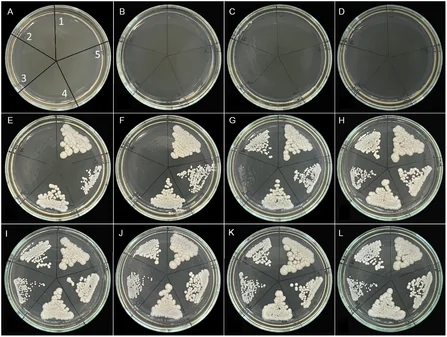
The results of the antifungal activity of essential oil from *Achillea biebersteinii* flowers using the agar dilution method. (1) *C. krusei*, (2) *C. glabrata*, (3) *C. parapsilosis*, (4) *C. tropicalis* and (5) *C. albicans*. The essential oil concentrations between the A and K plates range between 30–0.029 µL/mL. Plate L is the positive control.

Agar plates kept in an incubator overnight are shown in Figure 3. The agar dilution results were the same as the broth dilution results. No growth was observed in the plates with the highest essential oil concentration, and *Candida* species growth occurred starting from plate E. *C. krusei*, *C. tropicalis,* and *C. albicans* inoculated in the first, fourth, and fifth regions, respectively, started to grow from plate E. Therefore, the MIC value for these species is 3.75 µL/mL, which is the value in plate D. *C. glabrata* started to grow significantly, and *C. parapsilosis* started to grow slightly starting from plate G. Thus, the MIC value for *C. glabrata* and *C. parapsilosis* was determined as 0.937 µL/mL. As a result of the antifungal activity, all MIC values are shown comparatively in Table 3.

**TABLE 3:** The MIC values of the *Achillea biebersteinii* flowers’ essential oil and antifungal drugs against tested *Candida* strains.

Microorganisms	Essential oil MIC values, µL/mL	Ketoconazole MIC values, µg/mL	Tioconazole MIC values, µg/mL
*Candida krusei*	3.75	0.097	0.003
*Candida glabrata*	**0.937**	0.024	0.006
*Candida parapsilosis*	**0.937**	0.024	0.097
*Candida tropicalis*	3.75	0.078	0.781
*Candida albicans*	3.75	0.156	1.562

*Note*: Bold indicates the highest activity.

### 2.4 Molecular Docking Analysis Results

Docking studies were carried out with 14 compounds (essential oil content 1% and above) obtained from *A. biebersteinii* flowers. 5JLC and 7AGB PDB‐coded proteins were used as targets in docking studies. Docking scores of the compounds against 5JLC were calculated between −5.011 and −6.547 kcal/mol. The compounds with the highest affinity were determined as α‐terpineol (−6.547 kcal/mol), borneol (−6.446 kcal/mol), and *trans‐*Pinocarveol (−6.412 kcal/mol). Docking scores of the compounds against 7AGB were calculated between −3.117 kcal/mol and −5.400. 3‐Thujanone (−5.400 kcal/mol), α‐terpineol (−5.126 kcal/mol), and *trans‐*Pinocarveol (−5.106 kcal/mol) have the best binding scores against 7AGB. For the validation of molecular modeling studies, native ligands present in the crystal structures of 7AGB and 5JLC were removed, minimized, and redocked from the KB70 and 1YN structures, respectively. RMSD values were calculated as 1.0283 and 3.3274 Å. Docking scores of the compounds are given in Table 4. To determine whether the docking score differences reflected true affinity trends or were influenced by molecular size, ligand efficiency values (LE = −Δ*G*/HA) were calculated for all constituents and the co‐crystallised reference inhibitors, as shown in Table 5.

**TABLE 4:** Docking scores of essential oil from *A. biebersteinii* flowers aganist 7AGB and 5JLC.

Essential oil	5JLC, kcal/mol	5JLC RMSD	7AGB, kcal/mol	7AGB RMSD
(−)‐α‐Pinene	−5.662	0.0350	−4.041	0.0447
Camphene	−5.849	1.1171	−4.013	0.0772
3‐Ethyltoluene	−5.962	0.6837	−4.441	0.0531
*p*‐Cymene	−5.971	0.4126	−4.590	0.1926
Eucalyptol	−5.127	0.0465	−3.480	0.0673
3‐Thujanone	−5.515	1.4663	−5.400	1.1699
α‐Campholenal	−5.144	0.6648	−4.651	1.1417
*Trans‐*Pinocarveol	−6.412	1.1546	−5.106	1.1583
Camphor	−5.062	1.8958	−3.117	1.0644
*Cis*‐Chrysanthenol	−6.184	0.0759	−4.650	1.0530
Borneol	−6.446	1.7636	−4.303	1.7452
α‐Terpineol	−6.547	1.0103	−5.126	1.0707
*Trans*‐Chrysanthenyl acetate	−5.840	1.6064	−3.806	2.0839
α‐Chloropinacolone	−5.011	0.9827	−4.343	1.2602
KB70^a^	—	—	−6.438	3.3274
1YN^a^	−7.483	1.0283	—	—

a Native ligands in 5JLC and 7AGB. RMSD values are Glide‐reported ligand RMSD for the top‐ranked pose relative to the LigPrep input conformation; experimental‐pose RMSD is only applicable to redocked native ligands (KB70 and 1YN).

**TABLE 5:** Ligand efficiency (LE = −Δ*G*/HA) values of essential oil constituents and co‐crystallised reference inhibitors against *C. glabrata* CYP51 (5JLC) and *C. parapsilosis* Sapp1p (7AGB). HA: number of heavy atoms.

Compound	HA	LE (5JLC)	LE (7AGB)
3‐Ethyltoluene	9	0.662	0.493
α‐Chloropinacolone	8	0.626	0.543
*p*‐Cymene	10	0.597	0.459
α‐Terpineol	11	0.595	0.466
Borneol	11	0.586	0.391
Camphene	10	0.585	0.401
*Trans*‐Pinocarveol	11	0.583	0.464
(−)‐α‐Pinene	10	0.566	0.404
*Cis*‐Chrysanthenol	11	0.562	0.423
3‐Thujanone	11	0.501	0.491
α‐Campholenal	11	0.468	0.423
Eucalyptol	11	0.466	0.316
Camphor	11	0.460	0.283
*T* *rans*‐Chrysanthenyl acetate	14	0.417	0.272
1YN (reference, 5JLC)	49	0.153	—
KB70 (reference, 7AGB)	51	—	0.126

**FIGURE 4:**
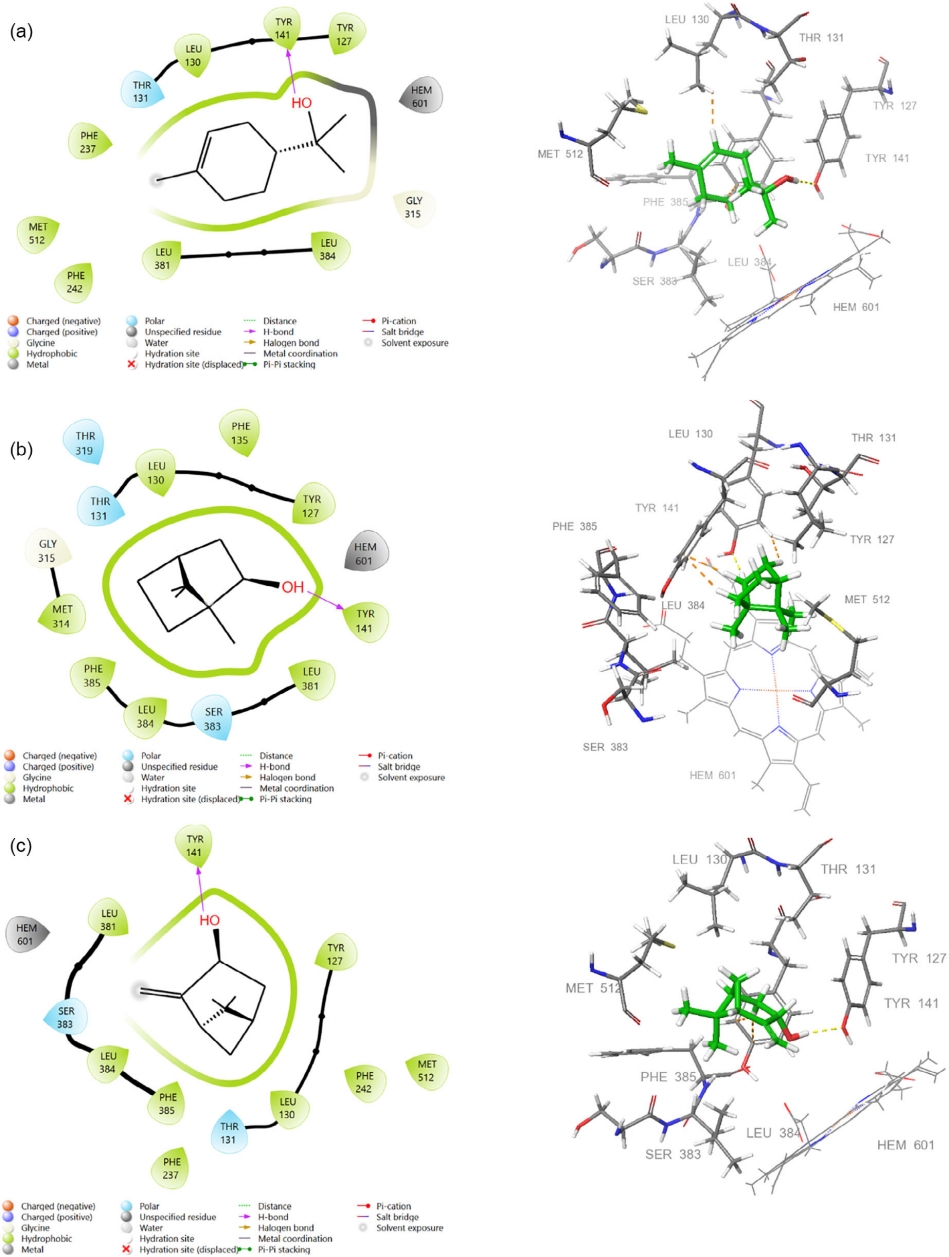
2D and 3D interactions of compounds in 5JLC. (a) α‐Terpineol, (b) borneol, (c) *trans‐*pinocarveol.

In order to determine the binding poses of the compounds to the target structures, the interactions of the compounds with the residues in the active site of the proteins were evaluated. In 5JLC, α‐terpineol formed hydrogen bonds with Tyr141 and polar interactions with Thr131. The compound also formed hydrophobic interactions with Tyr127, Leu130, Phe237, Phe242, Leu381, Leu384, and Met512. Borneol had neutral interactions with Thr131, Thr319, and Ser383, as well as hydrophobic interactions with Tyr127, Leu130, Phe135, Leu381, Leu384, Met314, and Phe385. *Trans*‐Pinocarveol, similar to α‐Terpineol and borneol, formed hydrogen bonds with Tyr141 and polar interaction with Thr131 and, Ser383. *Trans*‐Pinocarveol also made hydrophobic interactions with important residues, Tyr127, Leu130, Leu381, Leu384, Phe385, and Met512. The compounds formed hydrogen bonds via the hydroxyl (‐OH) group in their structures. The compounds made hydrophobic interactions with aliphatic rings. It was seen that the interaction with Tyr141 was crucial in binding to 5JLC (Figure 4).

**FIGURE 5:**
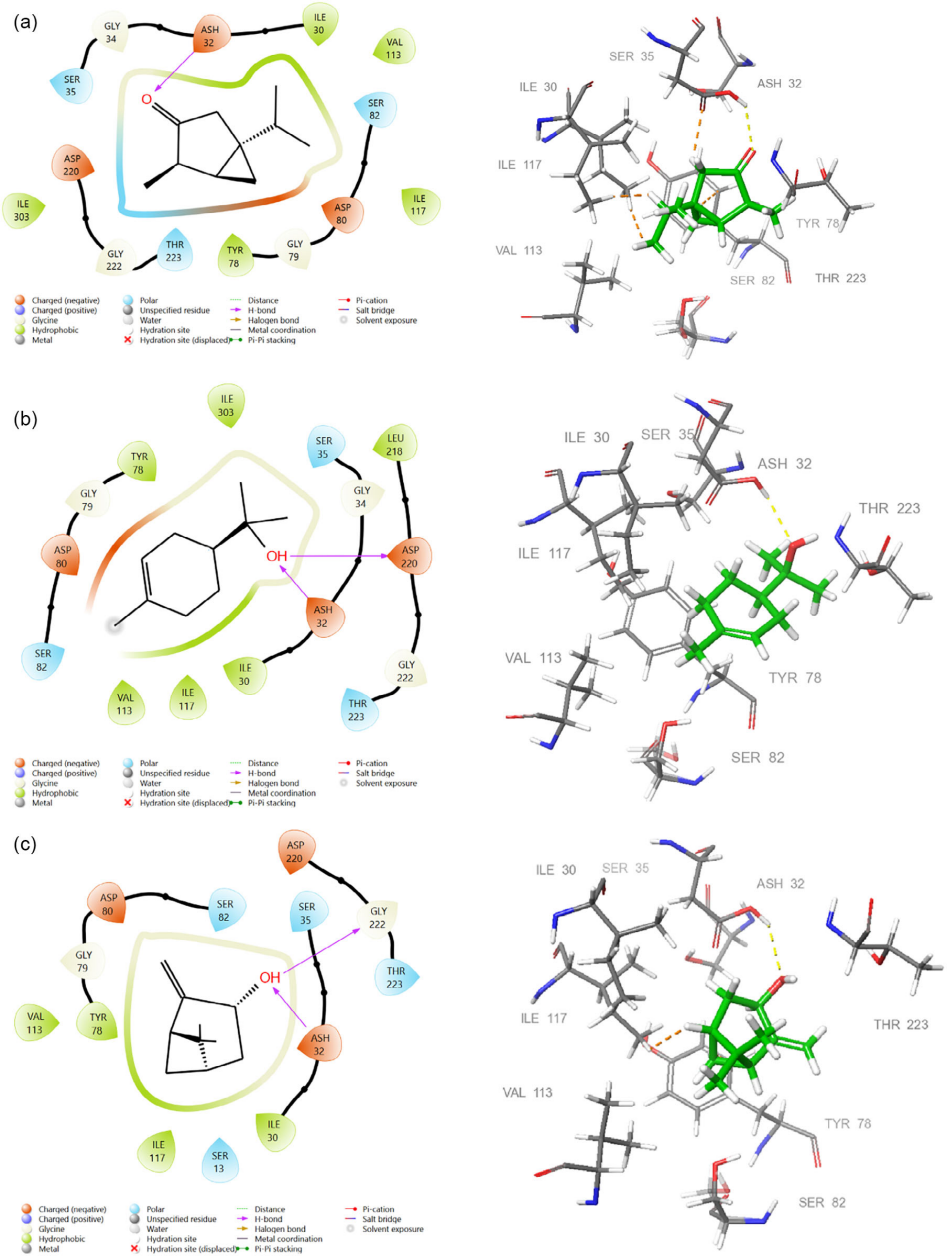
2D and 3D interactions of compounds in 7AGB. (a) 3‐Thujanone. (b) α‐Terpineol. (c) *trans‐*Pinocarveol.

The compound with the best binding affinity against 7AGB was 3‐Thujanone, which formed hydrogen bonds with Asp32 in the active site of the protein and formed charged (negative) interactions with Asp80 and Asp220. The compound formed polar interactions with Ser35, Ser82, and Thr223, and hydrophobic interactions with Ile30, Thr78, Val113, Ile117, and Ile303. Through its hydroxyl group, α‐terpineol made hydrogen bonds with Asp32 and Asp220 in the enzyme's active site. α‐Terpineol also formed polar interactions with Ser35, Ser82, and Thr223, and hydrophobic interactions with Ile30, Thr78, Val113, Ile117, Leu218, and Ile303. In the active site of 7AGB, it was observed that *trans*‐Pinocarveol, α*‐*terpineol, and 3‐Thujanone had similar interactions (Figure 5). The three compounds formed hydrogen bonds with Asp32, polar interactions with Ser35, Ser82, and Thr223, and hydrophobic interactions with Ile30, Thr78, Val113, and Ile117. Hydroxyl and ketone groups played an important role in the hydrogen bonding of compounds.

In 5JLC, the best‐scoring ligands adopted similar poses, where a single —OH group provided the main polar group (notably around Tyr141/Thr131), and the terpene ring system was stabilized by hydrophobic contacts in the pocket. In 7AGB, binding was concentrated around the catalytic acidic region, and —OH/ketone groups supported hydrogen bonding (especially with Asp32), while the nonpolar scaffold contributed additional packing. This indicates that 5JLC favors hydrophobic complementarity with limited polar groups, whereas 7AGB is more dependent on positioning oxygenated functions near the catalytic site, which is relevant when considering these constituents as dual‐target antifungal candidates.

## 3 Discussion

Essential oils form the basis of aromatherapy treatment [68]. Each essential oil has different effects on both physical and mental health depending on the compounds it contains and the chemotype distribution of these compounds. Antioxidant and antifungal activities on body health are among these effects. Chemical diversity in essential oils affects the pharmacological activities of the essential oil. Research indicates that these mixes are likely to demonstrate both synergistic and antagonistic effects, contingent upon the experimental settings [65]. In the present study, a turquoise‐green essential oil was obtained from the flowers of the plant with a yield of 0.87 µL/g. The essential oil, whose main components are 1,8‐cineol (eucalyptol; 21.84%), *cis*‐chrysanthenol (17.86%), camphor (15.29%), (−)‐α‐pinene (6.14%), and *trans*‐chrysanthenyl acetate (5.80%), showed low antioxidant properties with IC_50_ values of 11.76 and 3.45 mg/mL for DPPH• and ABTS^+^ radical scavenging, respectively. Although many investigations have examined *A. biebersteinii* essential oil^8^, only one investigation has focused on its antioxidant properties. The study by Sökmen et al. [69] reported that the main components of the essential oil obtained from the aerial parts of the plant, constituting 68.3%, were piperitone (34.9%), eucalyptol (13.0%), camphor (8.8%), chrysanthene (8.2%), and borneol (4.4%). The study reported that the essential oil had no potential antioxidant effect with an IC_50_ value of 4500 ± 45.00 µg/mL in the DPPH• test against curcumin, ascorbic acid, and butylated hydroxytoluene (BHT) standards, and also, these main components did not exhibit any antioxidant activity in the same test [69], implying that the antioxidant capacity reported for the whole essential oil in other studies was probably due to a synergistic interaction of the components rather than a single main compound. Compared to the present study, piperitone was completely absent among the major components of the essential oil, while eucalyptol was the most abundant compound. *Cis*‐Chrysanthenol and camphor followed with higher proportions. In the DPPH• test, the lower IC_50_ value obtained in the current study demonstrates the higher antioxidant activity of this essential oil compared to the previous study. Taking into account the differences of chemotypes, the *cis*‐Chrysanthenol and camphor with eucalyptol produced a sinergic effect, while the piperitone compound can be suggested to considerably antagonize the antioxidant effect of the essential oil. Based on these results, it can be thought that essential oils containing high levels of ketone derivative compounds, such as piperitone, may reduce antioxidant activity, while those rich in hydroxyl groups, such as phenolics and unsaturated terpenes, may enhance antioxidant activity.

The primary function of essential oils in plants is to protect the plant by acting as antimicrobial, antiviral, and insecticidal agents. The fatty acids present in essential oils can disintegrate the phospholipids in the cell membranes of mitochondria and bacteria. This action disrupts the membrane structures of microbial cells, making them more permeable. Consequently, the vital molecules and ions necessary for the survival of microorganisms infiltrate into the microbial cell, leading to cell death [70, 71, 72]. In this study, the high concentration of compounds in the plant essential oil directly affects their antifungal activity. As a result of GC–MS, eucalyptol has been found to be the most common compound in *A. biebersteinii* flowers’ essential oil, with a ratio of 21.84%. In another study, the antifungal activity of 1.8‐cineole, which is isolated from essential oil, was shown at 0.918 mg/mL of MIC, and the antifungal activity of essential oil (MIC: 0.227–0.908 mg/mL) was attributed to 1.8‐cineole, the most common compound [60]. In an antifungal activity study conducted by Morcia [61] and her colleagues, eucalyptol was tested against ten different yeast species, demonstrating antifungal activity; however, the mechanism of action was not elucidated in the study [61]. In another study, the antifungal efficacy of eucalyptol was determined at a concentration of 0.25 mg/mL (MIC) [73]. In this study, the MIC value of the plant flower essential oil against the tested *Candida* species ranged from 0.937 to 3.75 µL/mL. It is thought that eucalyptol, which is most commonly found in the fat content, enhances this activity.

In this study, the second most abundant compound in the flower essential oil was *cis*‐Chrysanthenol (17.86%). Studies on the antimicrobial activity of *cis*‐chrysanthemum have been conducted with plants containing *cis*‐chrysanthemum. The MIC value of the essential oil (containing *cis*‐Chrysanthenol between 6.7% and 7.6%) obtained from *Tanacetum vulgare* against dermatophyte strains was found to be 0.16–0.64 μL/mL [74]. In another study, *cis*‐Chrysanthenol, isolated from *Artemisia absinthium*, was found to have significantly promising antifungal activity against yeast strains *Fusarium moniliforme* and *Botrytis cinerea* [75]. Herewith, in this study, we can consider that *cis*‐Chrysanthenol, which is found in high levels in the essential oil, also significantly affects the antifungal results. In this study, camphor was found in plant flower oil at a rate of 15.29%. In a study where the antifungal activity of camphor was tested against four different species of *Fusarium* (the fungal phytopathogen), the MIC values of camphor against yeast species were observed to be 2–4 mg/mL [76]. The cell walls and cytomembrane structure of fungal cells treated with camphor are damaged, resulting in increased cell permeability. This increased permeability causes the leakage of microbial intracellular substrates, thereby disrupting the normal functioning of fungal cells [76]. Therefore, it can be thought that camphor contributes to the antifungal activity by disrupting the structure of *Candida* cell membranes in this study. The most abundant compound after camphor is α‐pinene at 6.14% in this work. Several studies in the literature have examined the antifungal properties of α‐pinene [77, 78, 79, 80]. One of these studies is related to the inhibitory activity of α‐pinene on *Candida* species. Minimum fungicidal concentration (MFC) values of α‐pinene against *C. albicans* and *C. parapsilosis* were found between 64 and 128 μg/mL [80]. α‐Pinene changed the profile of fungal structures and significantly reduced the number of pseudohyphae and blastoconids. Therefore, it was determined that α‐pinene could inhibit the morphological transition process of *Candida* cells from yeast to pseudohyphae, a key virulence factor [57, 80].

In this work, *trans‐*Chrysanthenyl acetate was found at a rate of 5.80%. Although there are not enough studies in the literature on the antifungal activity of pure *trans‐*Chrysanthenyl acetate, the antifungal properties of some plants have been attributed to the *trans‐*Chrysanthenyl acetate they contain [81, 82, 83]. In these studies, although no strong activity was observed against *Candida* species, the result was interpreted as suggesting that the substances in the plant content may have an antagonistic effect. Therefore, the strong antifungal activity of *A. biebersteinii* flowers’ essential oil can be attributed to the first four substances (Eucalyptol, *cis*‐Chrysanthenol, camphor, and α‐pinene) found more intensely in its content. Consequently, although the inhibitory effect of *A. biebersteinii* flowers’ essential oil on *C. glabrata* and *C. parapsilosis* was slightly more pronounced than that on *C. albicans*, *C. krusei*, and *C. tropicalis*, in general, the plant essential oil showed significant activity on *Candida* species.

Additionally, in this study, the antifungal MIC values of the flower essential oil were compared with those of ketoconazole and tioconazole, which were tested as controls (Table 3). The results demonstrate an acceptable difference in coefficients between the MIC values. Therefore, the plant essential oil, which has been shown to have antifungal activity, exhibited a more pronounced inhibitory effect on *C. glabrata* and *C. parapsilosis* than on the other *Candida* species tested, and the MIC values found were comparable to those of the control antifungals. This finding paves the way for further research and exploration into the specific mechanisms of the plant essential oil's antifungal activity.

Several studies in the literature have examined the antimicrobial activity of the essential oil obtained from *A. biebersteinii*. Sökmen and colleagues used well and disc diffusion methods to study the essential oil obtained from the aerial parts of *A. biebersteinii*. Inhibition of the plant's essential oil against the tested bacterial species and *C. albicans* was observed [69]. In another study, the antibacterial activity of the essential oil of *A. biebersteinii* was tested using the disc diffusion method; however, this method did not allow for the determination of the MIC values of the essential oil against these bacteria [84]. Almadiy and colleagues studied the antibacterial activity of the essential oil of *A. biebersteinii*. They found the MIC and MBC (Minimum Bactericidal Concentration) values of the oil to range from 60 to 480 μg/ml against the tested bacteria [85]. In another study, the essential oil of *A. biebersteinii* was tested alone or in combination with amikacin, along with other plants, against *Acinetobacter* isolates, and the oil was observed to inhibit *Acinetobacter* isolates [86]. Similarly, Kılıç et al. [87] and Al‐Shuneigat et al. [88] focused on investigating the antibacterial activity of the essential oil of *A. biebersteinii* [87, 88]. Mirahmadi and Norouzi tested the essential oil of *A. biebersteinii* against five different fungal species (*Aspergillus flavus*, *Rhizoctonia solani*, *Sclerotinia sclerotiorum*, *Fusarium verticillioides*, and *Bipolaris oryzae*) using the disc diffusion method. However, no MIC values were given against these tested microorganisms [89]. In another study, similar to ours, the inhibitory effect of the essential oil of *A. biebersteinii* on various *Candida* species and bacterial species was tested. However, the MIC values in the results were found to be between 15 and 62.5 μg/mL [90]. These values are high compared to our findings of MIC values (which ranged between 0.937 and 3.75 μL/mL). *A. biebersteinii* was collected from Ağrı (Eastern Turkiye) by Toplan and colleagues in 2017. Therefore, the phytochemical content of the plant may vary depending on the region where it grows and the time of collection [90].

It is stated that the contribution of all oxygenated monoterpenoids in the structure of essential oils and the synergistic interactions between these components play a decisive role in the strong antibacterial and antifungal activities they exhibit [91, 92]. The bioactive compounds contained in essential oils are directly responsible for the observed biological functions, and the mixtures formed by these compounds offer a much more significant and indicative antimicrobial potential than the effect shown by the individual main components [92, 93]. This situation can be explained by the synergistic mechanism created by the presence of oxygenated monoterpenoid molecules together [93, 94, 95, 96]. Research in the literature indicates that specific components such as 1,8‐cineol, cis‐chrysanthenyl acetate, camphor, and terpinen‐4‐ol clearly demonstrate remarkable antimicrobial properties [96, 97, 98]. Accordingly, the dominant antifungal activity highlighted in our study findings is thought to be related to the high concentration of these molecules in the plant matrix. However, this high activity may not be limited to the main components alone. Still, it may be the product of a collective synergy involving other secondary components present in the analyzed essential oil. Therefore, attributing the biological activity exhibited by a complex compositional system like an essential oil solely to a single molecule or a limited number of components is not a rational approach. Indeed, the presence of major or minor components in the mixture can directly or indirectly contribute to the observed antimicrobial profile. Furthermore, when evaluating the biological efficacy of essential oil matrices, a holistic approach is necessary to consider the potential synergistic and antagonistic interactions that compounds may have with each other in microbial inhibition processes. For these reasons, in future studies, isolating the complex mixture by separating it into fractions, purifying the resulting pure products, and independently testing the microbial growth inhibitory capacity of each component are of great importance in clarifying the mechanisms of action.

In molecular modeling studies, the enzyme structure with the code 5JLC pdb (structure of CYP51 from the pathogen *C. glabrata*) was used. The CYP51 enzyme, also known as lanosterol 14α‐demethylase, plays an important role in the conversion of lanosterol into ergosterol. Lanosterol is used in the biosynthesis of ergosterol, a basic component of fungal cell membranes [62, 70]. CYP51 inhibition disrupts ergosterol production and cell membrane integrity in fungi and causes cell death. Plant‐based compounds such as berberine target CYP51 inhibition and show broad‐spectrum antifungal activity against various fungal pathogens [63].

Protease Sapp1p from *C. parapsilosis* (7AGB) was determined as another target in modeling studies; it is an extracellular enzyme with broad substrate specificity that enables it to overcome barriers used to prevent invasive infections. Inhibition of the protease Sapp1p enzyme is an important target in antifungal activity studies against *Candida* species. Saps enhance yeast virulence by degrading host proteins involved in defense. They are also required for proper yeast adhesion during biofilm development. SAP enzymes contribute to virulence [64].

To integrate the in vitro findings with the in silico predictions, we compared the GC–MS abundance of the major constituents (Table 1) with their docking scores against 5JLC and 7AGB (Table 4). Against 5JLC, the strongest predicted compounds were α‐terpineol (−6.547 kcal/mol), borneol (−6.446 kcal/mol), and *trans*‐Pinocarveol (−6.412 kcal/mol); notably, the abundant constituent *cis*‐Chrysanthenol also showed a favorable score (−6.184 kcal/mol). In contrast, for 7AGB, the best scores were obtained with 3‐Thujanone (−5.400 kcal/mol), α‐terpineol (−5.126 kcal/mol), and *trans*‐Pinocarveol (−5.106 kcal/mol), whereas the major components eucalyptol (−3.480 kcal/mol) and camphor (−3.117 kcal/mol) showed comparatively weaker predicted binding. Since MIC values were determined for the essential oil mixture, this comparison is presented as composition‐guided prioritization rather than a direct compound–MIC correlation.

Modeling studies were conducted to determine the effects of compounds in the essential oil of *A. biebersteinii* flowers on the antifungal activity. In silico studies determined that α‐terpineol and *trans‐*Pinocarveol in the essential oil were two of the components with the highest affinity against both *C. glabrata* and *C. parapsilosis* and were determined to be potential antifungal compounds.

## 4 Conclusion

Essential oils, or aromatic oils, are mixtures mainly employed in cosmetics, though they also receive interest in pharmaceuticals due to their antimicrobial abilities. In this field, essential oils that offer health advantages by specifically inhibiting *Candida* species infections are of considerable significance. This study exhibits the advantages of the essential oil extracted from *A. biebersteinii* Afan. flowers using in vitro and in silico experiments. The essential oil contained 14 compounds, accounting for over 1% of 46 phytochemical substances. The essential oil demonstrated both modest antioxidant activity and exhibited considerable antifungal activity against *Candida* species due to terpene molecules. The main compounds of the essential oil, which exhibited significant affinity with the same MIC values against *C. glabrata* and *C. parapsilosis*, were assessed alongside molecular docking studies and in silico experimental findings. The high binding scores exhibited by α‐terpineol and *trans*‐Pinocarveol indicate that these compounds may be responsible for the antifungal activity. The therapeutic efficacy of essential oils containing these major compounds in treating candidal infections should be assessed in both the cosmetic and pharmaceutical fields. Subsequent research validating standardized essential oil of *A. biebersteinii* Afan. Through in vivo investigations, it will be a significant advancement for the scientific community.

## 5 Experimental Section

### 5.1 Plant Material and Preparation of Essential Oil

The aerial part of *A. biebersteinii* was collected, weighing almost 70 g, by Hilal Noyan at the time of flowering in June 2022 from the Aliceri village of Puturge/Malatya. The species was identified by Prof. Dr. Turan Arabacı (Voucher Specimen Code: ZT105). The plant was dried in a cool and shaded place and then stored for experiments. The flowers of the plant are used for the production of essential oil. 51.6283 g of flowers, cut into small pieces, was placed into a 1000 mL round‐bottom glass flask. After adding several boiling stones, 500 mL of distilled water was subsequently added. The sample flask was attached to a Clevenger apparatus, adhering to the European Pharmacopoeia standards, utilized for an essential oil lighter than water, and heated to elevated temperatures using an electric heater until boiling commenced. Subsequently, hydrodistillation proceeded at a moderate temperature for an average of 4 h. The yield of essential oil was determined volumetrically using a 0.01 mL‐graded 1 mL column of the Clevenger apparatus. The obtained essential oil was stored in a 1.5 mL amber‐colored bottle in *a* + 4 °C refrigerator.

### 5.2 Chemical Composition by GC–MS Analysis

The chemical composition of the essential oil from *A. biebersteinii* was determined by GC–MS at Atatürk University Eastern Anatolia High Technology Application and Research Centre (DAYTAM). Gas chromatography‐mass spectrometry (GC–MS) analysis was performed using a Shimadzu QP‐2010 Ultra (Ultra‐fast model, Japanese) gas chromatograph equipped with a flame ionization detector (FID) and a Restek GC Capillary Column (Rxi‐5 ms, 30 m, 0.25 mm ID, 0.25 µm). The oven temperature was held constant at 50 °C for 1 min and then programed at a rate of 6 °C/min to 300 °C and kept constant at this temperature for 15 min. The column oven temperature was 50 °C. The carrier gas was helium with a flow rate of 1 mL/min with a linear velocity of 25.4 cm/s, a split ratio of 1–25, and 0.30 s of event time. The reading started at 3 min and ended at 57 min, and the total ion current ranged from 40 to 400 m/z. The injection volume was 1 µL, the injector temperature was 280 °C, and the detector temperature was 310 °C. The percentages of compounds were measured using the area‐normalization method without considering response factors.

Compound characterizations were made solely based on comparisons of mass spectra with the NIST library. For quantifying purposes, relative area percentages derived from FID were utilized without correction factors.

### 5.3 Determination of Antioxidant Activity

#### 5.3.1 DPPH• Radical Scavenging Assay

This assay was modified based on the method presented by Shirwaikar et al. [99]. Methanol was used as a solvent for all stocks of essential oil and standard, and DPPH• solution. Methanol was also included as a control group. α‐Tocopherol was used as a reference standard. For this procedure, 0.0001 M DPPH• (2,2‐diphenyl‐1‐picrylhydrazyl, TCI) was prepared freshly. The essential oil (100, 75, 50, 30, 20, 15, 10, 7.5, 5, 2.5, 3.75, and 1.25 µL/mL) and α‐tocopherol (100, 80, 60, 40, 20, 10, 5, 2.5 µg/mL) were diluted using methanol. A 96‐well microplate was utilized in this study. Each well was filled with 50 µL of dilutions of samples and standards. Subsequently, 150 µL of freshly produced DPPH• reagent was introduced and incubated in the dark for 30 min at room temperature, 25 °C. Following incubation, absorbance readings were recorded at 517 nm. The experiments were performed in triplicate. The proportion of DPPH• inhibition was determined using the formula, as shown below. The half‐maximal inhibitory concentration (IC_50_) value was derived using the calibration curve. (ABS control = absorbance of the control group and ABS sample = absorbance of the sample used).

DPPH• scavenging effect %= [ABS control‐ABS sample]/ABS control×100

#### 5.3.2 ABTS^+^ Radical Scavenging Assay

The ABTS^+^ radical‐scavenging effect was altered as per the research conducted in previous studies [100, 101]. Stock 1 (2.45 mM potassium persulfate solution) and Stock 2 (7 mM ABTS^+^ solution) were formulated with distilled water. The ABTS^+^ solution was prepared by combining equal quantities of Stock 1 and Stock 2, which were allowed to react in an amber glass container in darkness at room temperature for 12–16 h. The ABTS^+^ solution was diluted with methanol to achieve an absorbance of 0.78 ± 0.25 at 734 nm. Methanol was also included as a control group. The essential oil (100, 75, 50, 30, 20, 15, 10, 7.5, 5, 2.5, 3.75, and 1.25 µL/mL), and α‐tocopherol which was used as a reference standard (100, 80, 60, 40, 20, 10, 5, 2.5 µg/mL), were diluted using methanol. 20 µL of the sample and 200 µL of ABTS^+^ solution were dispensed into 96‐well plates. Absorbance readings were recorded at 734 nm after of 6 min. The experiment was performed in triplicate. The findings were presented as IC_50_ values.

$${\mathrm{ABTS}}^{+}\;\mathrm{scavenging}\;\mathrm{effect}\;\%=[\mathrm{ABS}\;\mathrm{control}‐\mathrm{ABS}\;\mathrm{sample}]/\mathrm{ABS}\;\mathrm{control}\times 100$$

### 5.4 Determination of Antifungal Activity

#### 5.4.1 Tested Strains and Culture Media

Five different *Candida* species were used for their antifungal activity. These species: *Candida krusei* (ATCC 14243), *Candida glabrata* (ATCC 2001), *Candida parapsilosis* (ATCC 22019), *Candida tropicalis* (ATCC 13803), and *Candida albicans* (ATCC 14 053) were all obtained from the American Type Culture Collection (VA, USA). Sabouraud 4% glucose agar (Chemsolute, Renningen, Germany) and sabouraud broth (Biolife, Milan, Italy) were used as media.

#### 5.4.2 Application of the Broth Dilution Method

The dilution methods applied were the same as those used previously [102, 103, 104]. First, in the broth dilution method application, 30 µL of essential oil was dissolved in 250 µL of dimethylsulfoxide (DMSO, Sigma–Aldrich, Darmstadt, Germany) and distributed to the first wells of the microplate, and a twofold dilution was applied. Therefore, the amount of flower essential oil in the first well to the tenth well varied between 30, 15, 7.5, 3.75, 1.875, 0.938, 0.469, 0.234, 0.117, and 0.058 µL/mL, respectively. Consequently, the DMSO concentration varied between 250 and 0.488 µL/mL (Final DMSO volume was ≤0.1%) from the first well to the tenth well. Afterward, standard inoculums were prepared from the tested *Candida* species, and one µL of the standard inoculum was inoculated into each well of the microplate except for the negative control wells. Standard inoculums of *C. krusei* (A), *C. glabrata* (B), *C. parapsilosis* (C), *C. tropicalis* (D), and *C. albicans* (E) were inoculated into wells A through E on the microplate, respectively. Then, the microplate was incubated at 35 °C overnight. The next day, 15 µL of resazurin sodium salt (Sigma–Aldrich, Darmstadt, Germany) prepared at 0.015% (w/v) was added to each well, and the microplate was kept in the incubator for 3–4 h. Minimum inhibitor concentrations (MICs) were determined by observing the color change on the microplate.

#### 5.4.3 Application of the Agar Dilution Method

In the agar dilution method, 360 µL of essential oil was dissolved in 1000 µL of DMSO and added to 11 mL of sabouraud agar media, and a twofold dilution was applied. Thus, the amount of essential oil from the first plate to the eleventh plate varied between 30 and 0.029 µL/mL. Afterward, separate standard inocula of each *Candida* species were prepared as in the broth dilution method, and they were inoculated on the specified areas on the plates (1. *C. krusei*, 2. *C. glabrata*, 3. *C. parapsilosis*, 4. *C. tropicalis,* and 5. *C. albicans*) using 10 µL blue loops. All plates were kept in an incubator at 35 °C overnight, and the appearance of colonies on the plates was observed the next day.

### 5.5 Molecular Docking Analysis

The Maestro (Schrödinger 2023–4) program was used in all molecular docking studies. The structure of CYP51 from the pathogen *C. glabrata* (PDB ID: 5JLC) and protease Sapp1p from *C. parapsilosis* in complex with KB70 (PDB ID: 7AGB) were used as target proteins. Crystal structures of the proteins were downloaded from https://www.rcsb.org/. Protein structures were prepared with the Protein Preparation Wizard interface. Water molecules, ligands, and ions in the protein were removed from the structure; hydrogen atoms were added, and missing chains were added. The ionization and tautomeric states were created by Epik, whereas the proton orientations were determined by PROPKA at pH 7.0 ± 0.2, and ligand ionization/tautomeric states were generated with Epik at pH 7.0 ± 0.2 (Schrödinger default settings). The native ligands in the crystal structures of the proteins were taken as the center, and 20 Å sphere grid boxes were created with the Receptor Grid Generation module. 14 compounds (% content >1) determined by GC–MS analysis in essential oil from *A. biebersteinii* flowers were used in docking studies. SMILES codes of the compounds were taken from https://pubchem.ncbi.nlm.nih.gov; structures of the compounds were prepared with the 2D Sketcher of the Maestro program. Compound protonation states, tautomers, and low‐energy conformations were prepared using the LigPrep module. Optimization was performed using the OPLS4 force field. Compounds were docked to the active sites of proteins 50 times, and docking scores (kcal/mol) and protein‐ligand interactions were evaluated [105, 106, 107]. To validate the docking protocol, the co‐crystallized ligands were extracted and redocked into their respective binding sites using the same receptor grids and docking parameters. The redocked poses reproduced the crystallographic binding modes with heavy‐atom RMSD values of 1.0283 Å for 5JLC and 3.3274 Å for 7AGB (KB70).

## Author Contributions

Z.T. designed the research, performed the research, contributed data, analyzed data and wrote the paper. T.U. designed the research, performed the research, contributed data, analyzed data and wrote the paper. M.A.A. performed the research, contributed data, analyzed data and wrote the paper.

## Conflicts of Interest

The authors declare no conflicts of interest.

## Data Availability

The data that support the findings of this study are available from the corresponding author upon reasonable request.
